# Waist circumference a good indicator of future risk for type 2 diabetes and cardiovascular disease

**DOI:** 10.1186/1471-2458-12-631

**Published:** 2012-08-09

**Authors:** Reijo Siren, Johan G Eriksson, Hannu Vanhanen

**Affiliations:** 1 Health Centre of City of Helsinki, Helsinki, Finland; 2 Department of General Practice and Primary Health Care, University of Helsinki, Helsinki, Finland; 3 National Institute for Health and Welfare, Helsinki, Finland; 4 Helsinki University Central Hospital, Unit of General Practice, Helsinki, Finland; 5 Folkhalsan Research Center, Helsinki, Finland; 6 Vasa Central Hospital, Vasa, Finland; 7 The Social Insurance Institution of Finland (KELA), Helsinki, Finland

**Keywords:** Waist circumference, Type 2 diabetes, Cardiovascular disease, Middle-aged men

## Abstract

**Background:**

Abdominal obesity is a more important risk factor than overall obesity in predicting the development of type 2 diabetes and cardiovascular disease. From a preventive and public health point of view it is crucial that risk factors are identified at an early stage, in order to change and modify behaviour and lifestyle in high risk individuals.

**Methods:**

Data from a community based study was used to assess the risk for type 2 diabetes, cardiovascular disease and prevalence of metabolic syndrome in middle-aged men. In order to identify those with increased risk for type 2 diabetes and/or cardiovascular disease sensitivity and specificity analysis were performed, including calculation of positive and negative predictive values, and corresponding 95% CI for eleven different cut-off points, with 1 cm intervals (92 to 102 cm), for waist circumference.

**Results:**

A waist circumference ≥94 cm in middle-aged men, identified those with increased risk for type 2 diabetes and/or for cardiovascular disease with a sensitivity of 84.4% (95% CI 76.4% to 90.0%), and a specificity of 78.2% (95% CI 68.4% to 85.5%). The positive predictive value was 82.9% (95% CI 74.8% to 88.8%), and negative predictive value 80.0%, respectively (95% CI 70.3% to 87.1%).

**Conclusions:**

Measurement of waist circumference in middle-aged men is a reliable test to identify individuals at increased risk for type 2 diabetes and cardiovascular disease. This measurement should be used more frequently in daily practice in primary care in order to identify individuals at risk and when planning health counselling and interventions.

## Background

It is well known that an unhealthy and sedentary lifestyle is associated with an increased risk for obesity. Besides the well recognized cardiovascular disease (CVD) co-morbidities and risk factors including type 2 diabetes (T2D), hypertension, and dyslipidemia, obesity is also by itself one major CVD risk factor [1]. Abdominal obesity is a stronger risk factor than overall obesity – often expressed as body mass index (BMI) – for the future development of T2D and CVD [2-4]. From a preventive and public health point of view it is crucial that risk factors are identified at an early stage, in order to change and modify behaviour and lifestyle in high risk individuals.

Several screening procedures, tests and questionnaires are available to assess future risk for type 2 diabetes and cardiovascular disease [5,6]. In an often hectic primary care setting, however, the use of questionnaires is often too time consuming. One single measurement of abdominal obesity – i.e., waist circumference − is easy to perform and it is known to associate closely with type 2 diabetes and cardiovascular disease [7]. However, cut-off values for abdominal obesity predicting future type 2 diabetes and cardiovascular disease are known to be population specific [8,9]. The aim of the present study was to assess the predictive value of one single measurement of waist circumference, as an indicator of risk for type 2 diabetes and cardiovascular disease in middle-aged Finnish men.

## Methods

### Subjects

Helsinki Health Centre and Helsinki Heart District carried out a study to assess the prevalence of the metabolic syndrome (MetS) among middle-aged men in the city of Helsinki, between the years 2001 and 2003. The aim of that study, the MBO-project, was to create a screening system to make middle-aged men aware of their potential risk for type 2 diabetes and cardiovascular disease [10]. In year 2001 all men aged 40, 45, 50, and 55 years living in the north-east district of Helsinki were invited to participate in a type 2 diabetes and cardiovascular disease risk assessment visit at their local Health Center. Approval of the study protocol was obtained from the Epidemiological Ethics Committee of Helsinki and Uusimaa Hospital District. Each participant gave his written informed consent. During the appointment with the trained nurses the participants completed one type 2 diabetes (FINDRISC) and one cardiovascular disease (The Modified North Karelia project risk index) [5,6] questionnaire and they were interviewed about their lifestyle.

**Figure 1:**
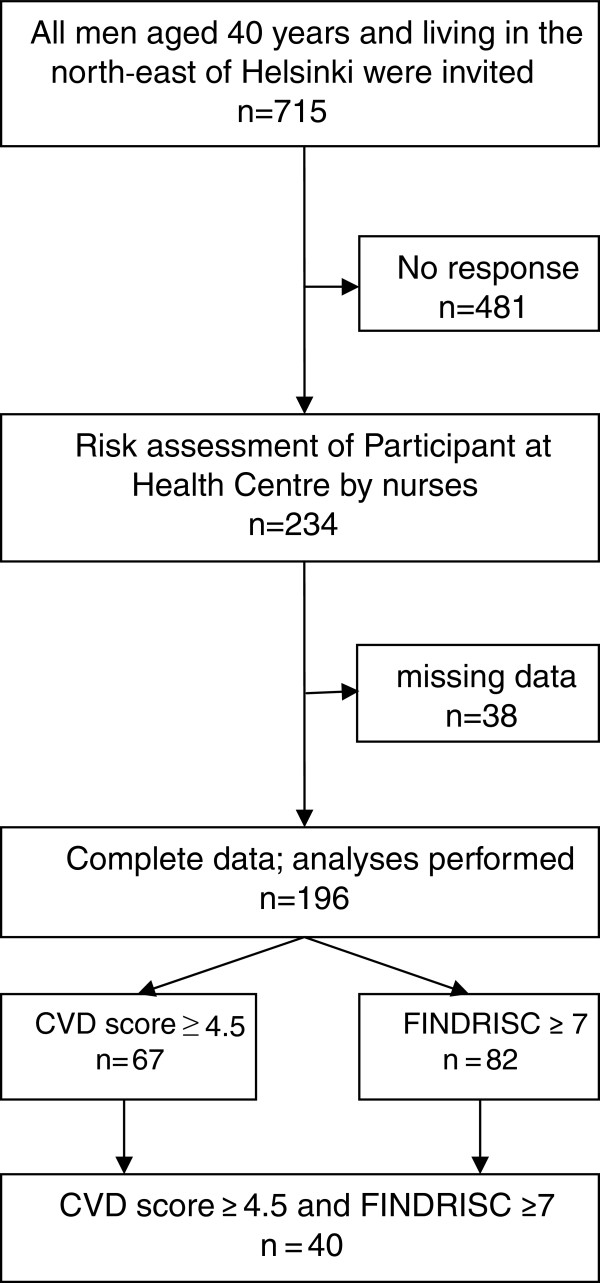
Flow chart and baseline characteristics of the study.

Blood pressure was measured in the sitting position, the mean of two measurements was used. Height was measured without shoes on to the nearest 0,1 cm, and weight was measured in light indoor clothing to the nearest 0.1 kg. BMI was calculated. Waist circumference was measured in the standing position, midway between the lowest rib and iliac crest, directly on the skin. All measurements were made by trained nurses according to standard techniques. Blood samples were drawn by a trained technician and analysed in a certified central laboratory for fasting lipids and glucose. Those with a CVD Risk Score of ≥ 4.5 received individualized lifestyle counselling based on their own risk profile. Those men at high risk had a follow-up visit 6 months later to evaluate the impact of the health counselling. Briefly, the observed reduction in CVD Risk Score was significant [10]. Based on the encouraging results of the original MBO project, Helsinki Health Centre is yearly inviting all men aged 40 years for risk evaluation and health-counselling visits. In the present study we focus upon men aged 40 years because they present the age group with the lowest morbidity and are potentially most suitable for preventive measures. Figure 1 shows the flow chart of the study and the number of men included.

### Questionnaires

The Diabetes Risk Score (FINnish Diabetes Risk Score – FINDRISC) has been developed at the National Public Health Institute of Finland and the validity of the test has been assessed by the same institute in an independent population survey [5]. The Diabetes Risk Score takes into account: age, BMI, waist circumference, exercise habits, dietary habits (intake of vegetables and berries), medication for elevated blood pressure, history of hyperglycemia, and family history of diabetes. A person with a risk score of 7 has at least 4 times higher risk to develop type 2 diabetes within 10 years than those whose risk score is below 7. The FINDRISC questionnaire can be accessed at http://www.diabetes.fi/english.

The CVD Risk Score: the modified version of the North Karelia project risk index is based on BMI, history of smoking, exercise habits, systolic and diastolic blood pressure, and total cholesterol concentration [6]. Depending on the risk factor status a person can have risk points from zero to sixteen. A person with at least 4.5 points is at high risk for cardiovascular diseases. The validity of the CVD Risk Score has recently been assessed [11].

### Analysis

We identified subjects with a risk score for type 2 diabetes ≥7 and subjects with a risk score for cardiovascular disease ≥4.5 from the study sample. Combining these two risk scores we identified subjects who had at least one of these risk scores elevated. The cut-off point for waist circumference was defined as the “positive test quantity”. Waist circumference was entered in the risk equation as a continuous variable. By cross tabulating frequencies of different categories we performed sensitivity and specificity analysis including positive and negative predictive values, and corresponding 95% CI for eleven different cut-off points (92 to 102 cm) of waist circumference. A ROC curve was plotted; sensitivity on the vertical axis and 1− specificity on the horizontal axis. When identifying frequencies and constructing the ROC curve we used the statistical program SPSS 16.0 for Windows (SPSS Inc., Chicago IL, USA). The sensitivity and specificity of the test with the positive, and the negative predictive values as well as the corresponding confidence intervals were calculated with the Confidence Interval Analysis (CIA) program for Windows according to Wilson’s method.

## Results

**Figure 2:**
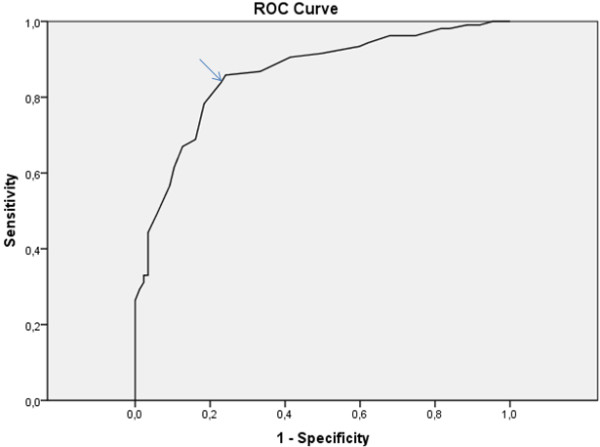
ROC curve.

Of the invited persons, 234 (32.7%) men participated, and provided baseline data. Waist circumference measurement of two men was missing. In the study sample median waist circumference was 95 cm and the range was 72 to 142 cm. Baseline characteristics of the study participants are shown in Table 1. The FINDRISC questionnaire was incomplete for 36 participants because the questionnaire was not yet available at the beginning of the MBO project. Both risk assessment tests were available for 196 men. Among these, 82 had a risk score for T2DM ≥7, and 67 had a risk score for CVD ≥4.5. At least one of the risk scores was elevated in 109 men, whereas both risk scores were elevated in 40. The percentiles, sensitivity, specificity, positive and negative predictive values of different cut-off points for waist circumferences are shown in Table 2. In this study population a cut-off point for waist circumference ≥94 cm identified subjects with increased risk for type 2 diabetes or for cardiovascular disease more precisely than any other cut-off point for waist circumference; sensitivity 84.4% (95% CI 76.6% to 90.0%), specificity 78.2% (95% CI 68.4% to 85.5%), positive predictive value 82.9% (95% CI 74.8% to 88.8%), and negative predictive value 80.0% (95% CI 70.3% to 87.1%), respectively. The sum of false positives (19/111) and false negatives (17/85) was smallest at the same cut-off point (≥94 cm). The area under the ROC curve was 0.857 (95% CI 0.805 to 0.909). The ROC curve is shown in Figure 2.

**Table 1:** General characteristics of the study sample

	**n**	**Mean**	**SD**	**Minimum**	**Maximum**
**Systolic BP mmHg**					
All	196	130.2	15.1	96	186
FINDRIC ≥ 7	82	133.6	14.8	110	186
CVD Risk Score ≥ 4.5	114	137.5	15.2	100	186
**Diastolic BP mmHg**					
All	196	86.2	9.6	65	120
FINDRISC ≥ 7	82	89.3	9.2	72	120
CVD Risk Score ≥ 4.5	114	92.5	10.1	66	120
**Total cholesterol mmol/l**					
All	196	5.06	1.08	2.8	8.2
FINDRISC ≥ 7	82	5.28	1.07	3.3	8.0
CVD Risk Score ≥ 4.5	114	5.88	1.02	3.5	8.3
**LDL cholesterol mmol/l**					
All	150	3.13	1.15	0.52	6.62
FINDRISC ≥ 7	71	3.24	1.06	0.52	6.38
CVD Risk Score ≥ 4.5	109	3.84	0.93	1.71	6.62
**HDL cholesterol mmo/l**					
All	150	1.44	0.45	0.78	2.59
FINDRISC ≥ 7	72	1.32	0.40	0.78	2.59
CVD Risk Score ≥ 4.5	110	1.33	0.35	0.76	2.67
**Triglycerides mmol/l**					
All	150	1.69	0.73	0.53	4.46
FINDRISC ≥ 7	72	1.84	0.88	0.54	4.46
CVD Risk Score ≥ 4.5	110	1.67	0.78	0.53	4.36
**Glucose mmol/l**					
All	150	5.37	0.59	4.2	8.2
FINDRISC ≥ 7	70	5.38	0.53	4.5	7.0
CVD Risk Score ≥ 4.5	111	5.45	0.48	4.3	7.0
**BMI kg/m**^**2**^					
All	196	26.2	3.8	18	44
FINDRIC ≥ 7	82	28.9	3.7	22	44
CVD Risk Score ≥ 4.5	114	28.1	4.0	18	44
**WC cm**					
All	196	96.6	10.4	76	142
FINDRISC ≥ 7	82	104.1	10.0	81	142
CVD Risk Score ≥ 4.5	114	101.5	10.4	82	142
**FINDRISC**					
All	196	5.8	3.6	0	18
FINDRISC ≥ 7	82	9.2	2.4	7	18
CVD RISK Score ≥ 4.5	82	4.8	2.7	0.5	11.5
**CVD Risk Score**					
All	196	3.8	2.6	0	18
FINDRISC ≥ 7	67	7.5	3.6	0	18
CVD Risk Score ≥ 4.5	105	6.7	1.8	4.5	12

BMI, body mass index; WC, waist circumference.

**Table 2:** Sensitivity, specificity, PPV and NPV corresponding to different waist circumference cut-offs

**Cut-off**	**Percentile**	**Sensitivity**	**Specificity**	**PPV**	**NPV**
**cm**		**%**	**%**	**%**	**%**
92	38.8	88.1	62.1	74.4	80.6
93	43.4	85.3	69.0	77.5	78.9
**94**	**47.4**	**84.4**	**78.2**	**82.9**	**80.0**
95	51.0	79.8	81.6	84.5	76.3
96	57.1	74.3	82.8	84.4	72.0
97	59.2	65.1	85.1	84.5	66.1
98	63.8	64.2	88.5	87.5	66.4
99	66.3	57.8	90.8	88.7	63.2
100	71.9	53.2	90.8	87.9	60.8
101	75.5	46.8	95.4	92.7	58.9
102	77.6	41.3	96.6	93.8	56.8

PPV, positive predictive value; NPV, negative predictive value.

## Discussion

Abdominal obesity, often expressed as an increased waist circumference, is becoming a widely accepted anthropometric measurement when assessing overall cardiometabolic risk. Several studies have shown that abdominal obesity correlates well with obesity related CVD risk factors including elevated blood pressure, dyslipidemia, and hyperglycemia [12,13]. Associations between waist circumference and type 2 diabetes and CVD-associated morbidity have also been demonstrated [14,15]. To our knowledge there is only one previous study focusing upon the association between waist circumference and future risk for type 2 diabetes/CVD assessing risk by applying a risk score equation [16]. From a public health point of view this is an important issue especially within primary care when aiming at early identification of high risk individuals. When estimating risk for CVD the CVD Risk Score compares well with SCORE relative risk chart [17]. The latter identified 87.6% of those with a CVD Risk Score ≥ 4.5 in our study, into the elevated risk category. Extrapolating those with CVD Risk Score ≥ 4.5 to 60 years of age in the SCORE risk chart, 88.6% had an absolute risk of ≥5%. These findings support the use of the CVD Risk Score as a screening tool in middle-aged men.

Waist circumference ≥ 94 cm is a compulsatory criterion for the metabolic syndrome (MetS) according to the criterion put forward by International Diabetes Federation (IDF). We applied IDF´s criteria to our study population and identified 21% of the men as having MetS and hence an increased risk of developing type 2 diabetes and/or CVD, whereas our method identifies 46% of the men from the same population with increased risk of developing type 2 diabetes and/or CVD. In the present study we demonstrated that among middle-aged men a cut-off point for waist circumference ≥94 cm detected with excellent precision those at increased risk of developing type 2 diabetes and/or CVD. The outcome variable; merged diabetes risk score and CVD Risk Score consist of a large number of cardio metabolic risk factors. By applying thresholds for diabetes risk score ≥7 and for CVD Risk Score ≥4.5, we avoided the possibility that only one single risk factor could lead to a positive outcome level. The highest single diabetes risk score (5 points) is obtained by an individual who at least occasionally have an elevated glucose level or who has a first-degree relative with diabetes. The highest single CVD Risk Score (4 points) is obtained by heavy smoking (at least 30 cigarettes per day) or by having a total cholesterol ≥ 8.5 mmol/l. Beginning in adolescent the risk characteristics accumulates little by little depending on the lifestyle. Already at the age of 15 years; boys have a significantly higher risk for CVD with WC ≥75^th^ percentiles compared those with WC ≤25^th^ percentiles [18]. Restoring to the former condition is more and more difficult in societies of today. In a 5 years follow-up study only overweight younger middle-aged men benefit from weight reduction regarding major CVD events whereas weight loss was associated with significant risk reduction in all age-groups regarding type 2 diabetes [19]. As the outcome variable is set to relative low level the test can identify subjects in the phase where the modification of lifestyle still has an impact on major risk factors. The measurement of waist circumference is an easy way to get reliable information of the risk for type 2 diabetes and CVD in the often hectic primary care settings. When the waist circumference measurement gives an alarm the practitioner has a method for the risk assessment for type 2 diabetes and for CVD to determine whether the alarm calls for lifestyle intervention. The main weakness of this study was the relatively small study sample. As we studied only Finnish men aged 40 years it is not possible to generalise the results into other age-groups. Further studies are also needed to determine cut-offs for women and for men other than Caucasian [20]. We demonstrated the capability of the “two phase screening method” to identify people at an increased risk for type 2 diabetes and for cardiovascular disease. This method is easy to apply in general practice. Implementation of the method is potentially challenging in the primary health care setting due to the rapid grow in prevalence of obesity. Therefore, it is important to incorporate the whole team and involve public health nurses to participate in identification and prevention of type 2 diabetes and cardiovascular disease.

## Conclusions

We conclude that a waist circumference above 94 cm was found to be most predictive in identifying middle-aged men having a high risk to develop type 2 diabetes or cardiovascular disease. It should therefore be used more in every day practice to identify individuals at risk.

## Competing interests

The authors declare that they have no competing interests.

## Authors’ contributions

RS, JGE and HV participated in the design of the study. RS performed the statistical analyses and wrote the first draft of the manuscript. All authors read and approved the final manuscript.

## Funding

The original MBO-project was supported by Finland´s Slot Machine Association (RAY).

## Pre-publication history

The pre-publication history for this paper can be accessed here:

http://www.biomedcentral.com/1471-2458/12/631/prepub
